# Topological Fractionation of Resting-State Networks

**DOI:** 10.1371/journal.pone.0026596

**Published:** 2011-10-19

**Authors:** Ju-Rong Ding, Wei Liao, Zhiqiang Zhang, Dante Mantini, Qiang Xu, Guo-Rong Wu, Guangming Lu, Huafu Chen

**Affiliations:** 1 Key Laboratory for Neuroinformation of Ministry of Education, School of Life Science and Technology, University of Electronic Science and Technology of China, Chengdu, People's Republic of China; 2 Department of Medical Imaging, Nanjing Jinling Hospital, Clinical School, Medical College, Nanjing University, Nanjing, People's Republic of China; 3 Laboratory of Neuro-psychophysiology, K. U. Leuven Medical School, Leuven, Belgium; Indiana University, United States of America

## Abstract

Exploring topological properties of human brain network has become an exciting topic in neuroscience research. Large-scale structural and functional brain networks both exhibit a small-world topology, which is evidence for global and local parallel information processing. Meanwhile, resting state networks (RSNs) underlying specific biological functions have provided insights into how intrinsic functional architecture influences cognitive and perceptual information processing. However, topological properties of single RSNs remain poorly understood. Here, we have two hypotheses: i) each RSN also has optimized small-world architecture; ii) topological properties of RSNs related to perceptual and higher cognitive processes are different. To test these hypotheses, we investigated the topological properties of the default-mode, dorsal attention, central-executive, somato-motor, visual and auditory networks derived from resting-state functional magnetic resonance imaging (fMRI). We found small-world topology in each RSN. Furthermore, small-world properties of cognitive networks were higher than those of perceptual networks. Our findings are the first to demonstrate a topological fractionation between perceptual and higher cognitive networks. Our approach may be useful for clinical research, especially for diseases that show selective abnormal connectivity in specific brain networks.

## Introduction

Exploring topological properties of the human brain network provides information about its organization and function [1], [2]. The human brain network has been widely demonstrated to have optimized small-world architecture [3], [4] in structural and functional domains, and at multiple temporal and spatial scales [5], [6], [7], [8], [9], [10], [11], [12]. The small-world architecture not only reflects brain functional organization principles of local specialization and global integration [13], but also maximizes the efficiency of information processing at a low wiring cost [4]. Small-world topology might reflect a general organization principle of human brain at either large-scale or voxel-scale level [14], [15], [16].

Resting-state networks (RSNs) derived from resting-state functional magnetic resonance imaging (fMRI) data provided evidence for a large-scale organization of intrinsic spontaneous brain activity [17], [18] into systems related to sensory, motor, language, social-emotional, and cognitive functions [19], [20], [21], [22], [23], [24], [25]. Previous task-related studies suggested functional fractionations of the brain, and in particular a dichotomy between lower-level perceptual (e.g., visual, auditory and somato-motor) and higher-level cognitive networks (e.g., attention, central-execution, and default-mode) during active behavior [19], [26], [27], [28], [29]. Different anatomical and connectional properties may partially explain the functional differences observed between these groups of networks [30], [31]. For example, Zielinski *et al.* revealed by structural covariance MRI techniques different development trajectories between perceptual networks and higher cognitive networks across developmental stages [32].

It is still unclear whether the dichotomy between lower-level perceptual and higher-level cognitive networks can be observed in resting state data. An increasing number of studies have focused on interactions between RSNs [33], [34], [35], documenting that each of them is involved in different levels of processing [31], [36]. Interestingly, Jann *et al.* revealed putative psycho-physiological dissimilarities between these two groups of networks, as reflected in the pattern of correlations between RSN time-courses and EEG power fluctuations [37].

Both structural and functional brain networks exhibit optimized small-world architecture at the whole-brain level. However, little experimental evidence exists for the topological properties of single RSNs. In the present study, we have two hypotheses: i) each RSN has optimized small-world architecture; ii) perceptual and higher cognitive networks have different network organization properties. On the basis of the aforementioned hypotheses, we first identified the RSNs using independent component analysis (ICA) and further applied graph theoretical analysis to investigate the topological properties of RSNs.

## Materials and Methods

### Subjects

A total of 38 right-handed healthy volunteers (20 females, age: 19–26 years) were recruited in this study. All subjects had no history of neurological disorder or psychiatric illness, and no gross brain abnormalities. Before MRI scanning, written informed consents was obtained from all the participants. The study was approved by the local medical ethics committee in Jinling Hospital, Nanjing University School of Medicine.

### Image acquisition

Imaging data collection was performed with a SIEMENS Trio 3T MR scanner (Erlangen, German) at Jinling Hospital, Nanjing, China. During data acquisition, the subjects were instructed to keep their eyes closed, relax, and remain as motionless as possible. Foam pads and earplugs were used to reduce head motion and attenuate scanner noise, respectively. Functional data were collected by using a single-shot, gradient-recalled echo planar imaging (EPI) sequence (TR = 2000 ms, TE = 30 ms and flip angle = 90°). Thirty transverse slices (FOV = 240×240 mm^2^, in-plane matrix = 64×64, slice thickness = 4 mm, inter-slice gap = 0.4 mm, voxel size = 3.75×3.75×4 mm^3^), aligned along the anterior commissure-posterior commissure (AC-PC) line were acquired. For each subject, a total of 255 volumes were acquired and the first five volumes were discarded to ensure steady-state longitudinal magnetization. Subsequently, high-resolution T1-weighted anatomical images were acquired in the sagittal orientation using a magnetization-prepared rapid gradient-echo (MPRAGE) sequence (TR = 2300 ms, TE = 2.98 ms, flip angle = 9°, FOV = 256×256 mm^2^, matrix size = 256×256 and zero filled and interpolated to 512×512, slice thickness = 1 mm, without inter-slice gap, voxel size = 0.5×0.5×1 mm^3^, and 176 slices).

### Data preprocessing

Preprocessing of functional images was carried out using the SPM8 software (http://www.fil.ion.ucl.ac.uk/spm). First, the 250 volumes were corrected for the temporal difference in acquisition among different slices; then, they were realigned to the first volume for head-motion correction. No dataset was excluded according to the criteria that head motion was less than 1.5 mm of displacement or 1.5 degree of rotation in any direction. Next, the realigned images were spatially normalized to the Montreal Neurological Institute (MNI) echo-planar imaging template and re-sliced to 3-mm cubic voxels. Then, they were spatially smoothed by convolution with an isotropic Gaussian kernel (FWHM = 8 mm) to attenuate spatial noise.

### Independent component analysis

Group spatial ICA was performed using the GIFT software (http://icatb.sourceforge.net/, version 1.3 h) [38]. First, the optimal number of independent components (ICs) was estimated to be 35 using the minimum description length (MDL) criterion [39], [40]. Then, fMRI data from all subjects were concatenated and the temporal dimension of this aggregate data set was reduced to 35 by using principal component analysis (PCA). ICs were estimated using the FastICA algorithm [41]. IC time-courses and spatial maps for each subject were back-reconstructed, using the aggregated components and the results from the data reduction step [38], [39].

### RSN identification

Six ICs corresponding to the RSNs of auditory, somato-motor, visual, central-executive, dorsal attention, default mode networks were selected using spatial-template correlation analysis [39], [42]. Specifically, our selected RSNs corresponded to the cerebral ICs with the largest spatial correlations with the network templates from our previous studies [25], [34], [43], [44]. The ICs corresponding to six RSNs were also extracted from single subject. A random-effect analysis was calculated on the spatial maps of corresponding RSNs from single subject, by using one-sample t-tests. Thresholds were set at p<0.01 (corrected for multiple comparison using the FWE criterion).

### Voxels time-courses extraction

For each subject, we extracted time-courses from gray matter voxels belonging to each RSN. Since RSNs partly overlap [22], [45], we excluded voxels belonging to more than one RSN from subsequent analyses. The voxel time-courses were first processed by linear regression to remove several sources of spurious variance and their temporal derivatives: (1) six motion parameters obtained by rigid body head motion correction, (2) white matter signal averaged from white matter, (3) ventricular signal averaged from ventricles, and (4) global brain signal averaged across gray and white matter voxels. The residuals of these regressions were temporally band-pass filtered (0.01–0.08 Hz) to reduce low-frequency drift and high-frequency noise, related to respiratory and other physiological processes [10], [11], [46].

### Power spectrum analysis of RSNs time-courses

Fourier power spectrum analysis on the entire frequency range (0–0.25 Hz) was performed for the averaged time-courses within each RSN in each subject. We calculated the relative power by dividing the power spectrum by its maximum value. We defined the contribution in the low-frequency bandwidth (0.01–0.08 Hz) as the ratio of the energy in this low-frequency range compared to that in the entire frequency range.

### Correlation matrix and functional network construction

To measure the voxel-level functional connectivity of each RSN [47], we calculated the Pearson correlation coefficients between the time-courses of RSN voxels. We thresholded the resulting correlation matrix to obtain an undirected binary graph (network). For a voxel-level network, a node represents a voxel, whereas an edge represents a link between voxels. Next, we analyzed the constructed networks using graph theoretical approaches.

### Graph theoretical analysis

**Topological properties of the voxel-level functional brain connectivity networks.** The topological properties of the functional brain connectivity networks were defined on the basis of a *N*×*N* (*N* represents the number of voxels in a RSN and is different in different RSN) binary graph, *G*, consisting of nodes and undirected edges:



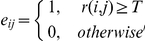
where 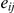 refers to the undirected edge between node 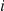 and node 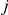 in the graph. In general, if 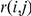 (Pearson correlation coefficient) of a pair of nodes, 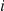 and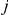, exceeds a given threshold 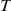, an edge is said to exist; otherwise it does not exist. A subgraph 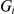 is defined as the graph including the nodes that are the direct neighbors of the 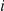
th node, i.e. directly connected to the 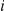
th node with an edge. The degree of each node, 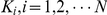, is defined as the number of nodes in the subgraph 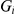.

The clustering coefficient of a node depicts the level of connectedness of the direct neighbors of this node. The clustering coefficient 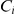 of voxel 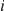 is defined as the ratio of the number of actually existing connections to the number of all possible connections in the subgraph 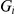:



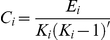
where 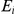 is the number of edges in the subgraph 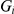. The clustering coefficient of a network is the average of the absolute clustering coefficient over all voxels in the network [4], [48]:



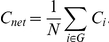




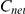
 is a measure of the extent of the local efficiency or cliquishness of information transfer on the network.

The mean shortest path length of a node is:
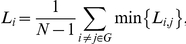
where 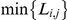 is the shortest path length between node 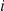 and node 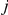, and the path length is the number of edges included in the path connecting two nodes. The mean shortest path length of a network is the average of the shortest path lengths between the nodes:



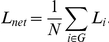




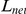
 is a measure of the extent of global efficiency or the capability for parallel information propagation of the network.

In a network, 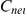 and 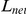 are key characteristics, and permit to define whether the network is a random network or small-world network. Random networks are characterized by a low clustering coefficient 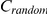 and a typical shortest path length 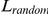. Compared with random networks, small-world networks have similar shortest path lengths but higher clustering coefficients, that is 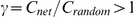, 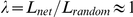 [4]. These two conditions can also be summarized into a scalar quantitative measurement, small-world-ness, 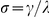, which is typically 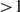 for networks with a small-world organization [10], [11], [49], [50]. To examine small-world properties, the value of 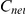 and 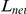 of the functional connectivity network need to be compared with those of a random network (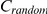 and 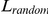). The theoretical values of these two measures for random networks are 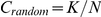, and 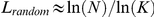 [3], [49], [51]. However, as suggested by Stam *et al.* [51], statistical comparisons should generally be performed between networks that have equal (or at least similar) degree sequences; however, theoretical random networks have Gaussian degree distributions that may differ from the degree distribution of brain networks. To obtain a better control for the functional brain networks [11], we generated 30 random networks for each individual network keeping the same degree for each node by using a Markov-chain algorithm [52], [53]. This procedure was repeated until the topological structure of the original matrix was randomized, resulting in a random graph with a degree distribution similar to that of the original matrix. We then averaged all 30 generated random networks to obtain mean values of 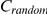 and 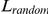.

#### Computation of network properties

The network topological properties are influenced by the choice of a threshold value. Conservative thresholds (increasing correlation coefficient threshold 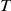) will generate sparsely connected graphs; more lenient thresholds (decreasing correlation coefficient threshold 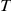) will generate more densely connected graph. Since there is currently no formal consensus regarding selection of thresholds, here we selected a range of correlation coefficient threshold (0.125≤*T*≤0.55, step = 0.025) for exploring the topological properties of functional connectivity graphs in all RSNs. The minimum correlation coefficient threshold 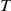 was chosen to exclude weak and potentially non-significant connections. Thus, it was set to 0.125, corresponding to *p*<0.05 (uncorrected) in the voxel-level correlation matrix. The maximum 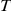 was empirically set to 0.55, ensuring that the largest subgraph included at least 90% voxels in the networks over all six RSNs and all subjects [10]. For each subject, the characteristics 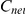 and 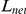 from the functional connectivity graph of each RSN were computed for different 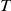. In order to compare accurately the topological properties of functional connectivity graphs among RSNs, we calculated 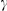, 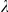 and the small-world index 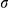 at the conservative threshold 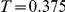
(corresponding to *p*<0.05, Bonferroni-corrected). This correlation coefficient threshold was used to reduce the chance of false positive connections of voxel-level correlation matrix across all six RSNs and all subjects. Generally, RSNs with different number of nodes may result in different network topological properties. To eliminate this potential confounding effect, we tested the sensitivity of the results (see Text S1 for detailed analysis).

## Results

### RSNs identification

**Figure 1:**
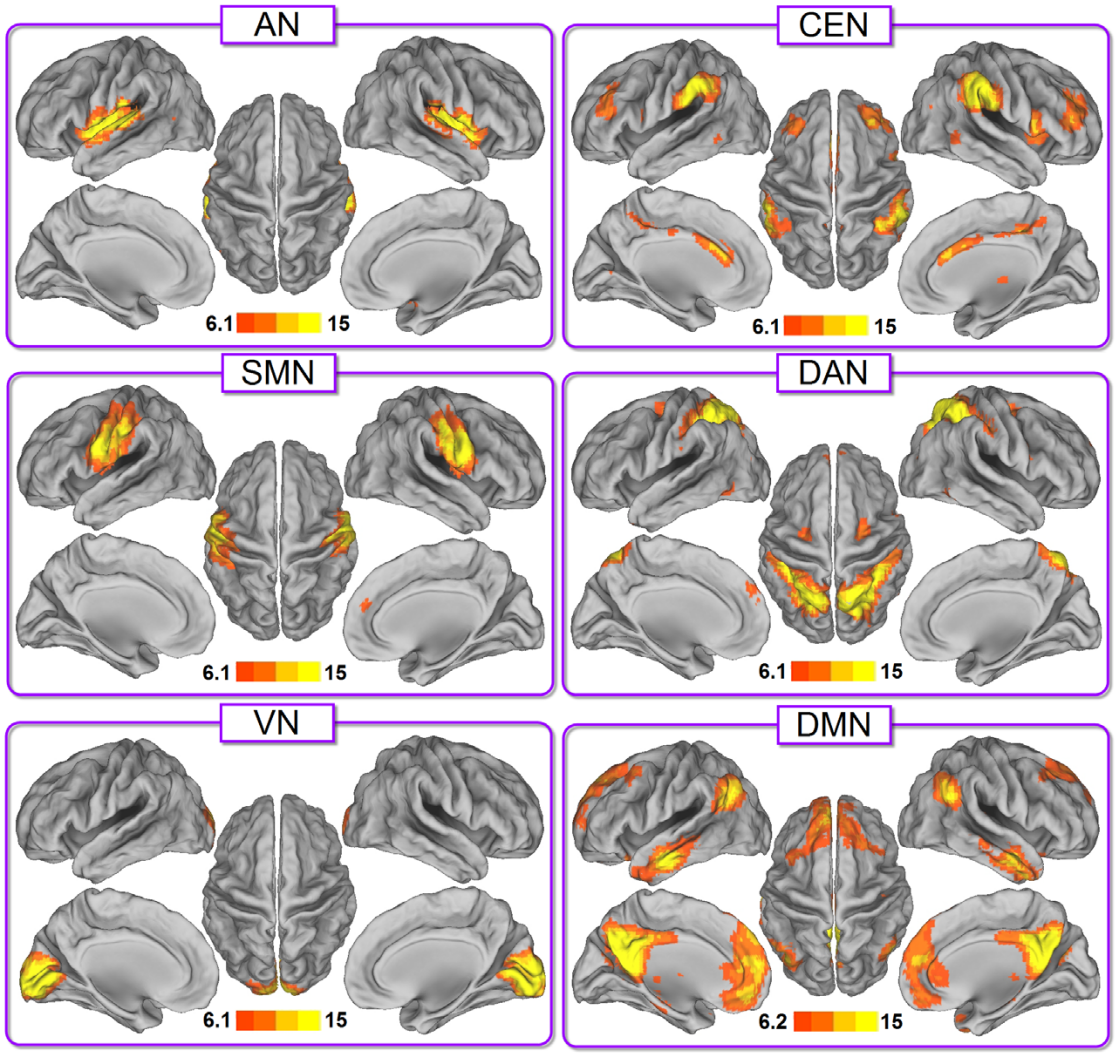
Cortical representation of the six RSNs. For each RSN, *Left*: lateral and medial views of left hemisphere; *Center*: dorsal view; *Right*: lateral and medial views right hemisphere.

The spatial maps of the six selected RSNs were obtained using the group spatial ICA analysis implemented in the GIFT software (http://icatb.sourceforge.net/, version 1.3 h) [38]. These were retrieved by means of a spatial-matching procedure. The RSNs, illustrated in Fig. 1, can be described as follows: 1) the auditory network (AN) primarily encompassed the bilateral middle and superior temporal gyrus, Heschl gyrus, insular cortex, and temporal pole; 2) the somato-motor network (SMN) included pre- and postcentral gyrus; 3) the visual network (VN) included the inferior, middle and superior occipital gyrus, and temporal-occipital regions along with superior parietal gyrus; 4) the central-executive network (CEN) included the dorsal lateral prefrontal and the posterior parietal cortices; 5) the dorsal attention network (DAN) primarily involved middle and superior occipital gyrus, parietal gyrus, inferior and superior parietal gyrus, as well as middle and superior frontal gyrus; 6) the default mode network (DMN) encompassed posterior cingulate cortex, bilateral inferior parietal gyrus, angular gyrus, middle temporal gyrus, superior frontal gyrus and medial frontal gyrus. On the basis of previous studies [31], [32], [34], [37], we partitioned the six RSNs into two groups: higher cognitive networks (CEN, DAN and DMN) and perceptual networks (SMN, AN and VN).

### Power spectrum of RSNs

**Figure 2:**
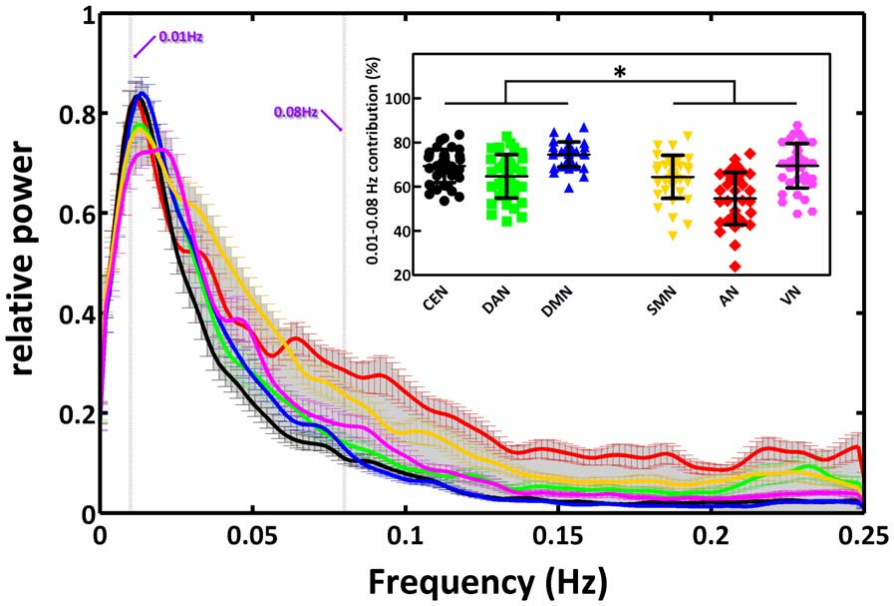
Spectral analysis of the RSNs' time-courses. (Main frame) Mean relative spectral distribution of the voxel-averaged time-courses of each RSN. (Embedded frame) Power contribution in the low-frequency band (0.01–0.08 Hz) for each RSN. Error bars correspond to SD. Asterisks indicate statistically significant differences between the groups of higher cognitive and perceptual networks (two-way ANOVA, p<0.01, Bonferroni-corrected).

The relative power and the power contribution of the low-frequency band (0.01–0.08 Hz) on the entire frequency range for each RSN are shown in Fig. 2. The power contribution in the low-frequency band was largest for the DMN, following VN, CEN, DAN, SMN and AN. We found a significant difference across RSNs in the low-frequency band, as assessed by a one-way analysis of variance (ANOVA) (*p*<0.01, Bonferroni-corrected). The higher level cognitive networks showed significantly greater low-frequency power than the perceptive networks (two-way ANOVA, p<0.01, Bonferroni-corrected) (Fig. 2). The measure of the power contribution in the low-frequency band corresponded to that of fractional amplitude of low-frequency fluctuations (fALFF) [45], [54]. We found indeed that higher cognitive networks exhibited higher fALFF than perceptual networks (Fig. S1).

### Clustering coefficient and shortest path length

**Figure 3:**
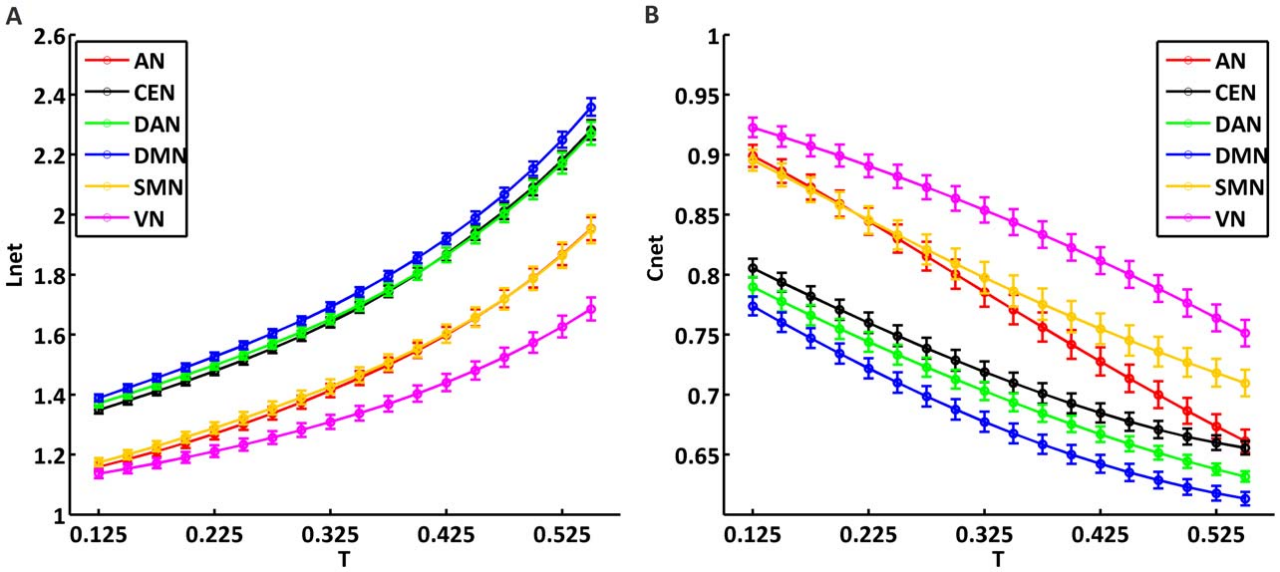
Mean path length and clustering coefficient for each RSN. (A) Shortest path length, 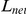, and (B) clustering coefficient, 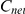, for each RSN as a function of correlation threshold 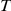 (0.125–0.55). Error bars correspond to SEM.

The average shortest path length (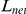) and their dependence on correlation coefficient threshold 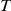 for each RSN are illustrated in Fig. 3A. As expected, 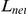 increased for larger values of the correlation coefficient threshold 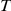, due to an increased number of paths. Over the range of threshold values (0.125–0.55), we observed similar 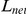 values among higher cognitive networks (CEN, DAN and DMN), and among perceptual networks (SMN, AN and VN). The values of the former group were significantly larger than those of the latter group (two-way ANOVA, *p*<0.01, Bonferroni-corrected). The average clustering coefficient (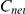) and their dependence on correlation coefficient threshold 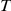 for the voxel-level functional network in each RSN are illustrated in Fig. 3B. 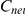 decreased for larger values of the correlation coefficient threshold. As for 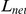, 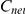 values of higher cognitive networks were clearly lower than those of perceptual networks. In summary, both 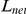 or 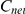 confirmed a dichotomy between the two groups of networks. To assess the sensitivity of the results with respect to the number of nodes in each RSN, we recomputed 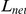 and 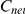 for RSNs with equalized number of nodes (Fig. S2). Importantly, we observed results similar to those obtained without the node-normalization procedure.

### Statistical analysis of network properties

**Figure 4:**
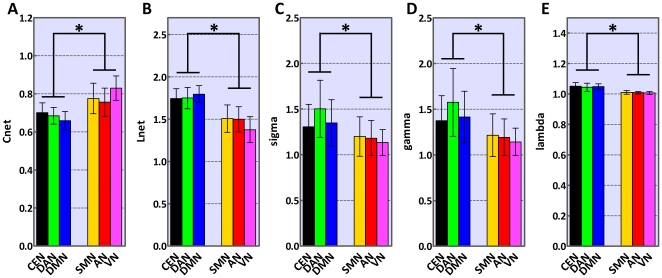
Statistical analysis of network properties for higher cognitive and perceptual networks. The group of higher cognitive networks includes DMN, DAN and CEN; the group of perceptual networks includes SMN, VN and AN. Binary graphs were calculated using the correlation threshold 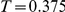. (A) Mean clustering coefficient, 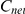; (B) shortest path length, 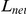; (C) small-world index, 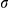; (D) normalized clustering coefficient, 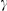; (E) normalized shortest path length, 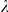. Error bars correspond to SD. Asterisks indicate statistically significant differences between the two network groups (two-way ANOVA, p<0.01, Bonferroni-corrected).

After the analysis of 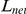 and 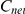 at different correlation coefficient threshold 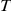, we examined in more detail network properties for each RSN using the correlation coefficient threshold 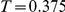. In particular, we calculated 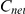, 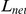, 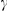, 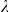 and the small-world index 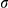. The related results are shown in Fig. 4 and Table 1. Both 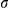 and 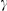 were significantly larger than 1 for each RSN and 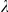 was not different from 1 (*p*<0.01, Bonferroni-corrected), suggesting a small-world organization (Table 1). We used a one-way ANOVA was used to test for significant differences of 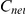, 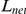, 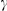, 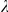 and 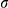 across RSNs; then, we tested the differences between the two network groups (higher cognitive vs. perceptual) by a two-way ANOVA. As shown in Fig. 4, 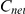 of higher cognitive networks was significantly lower than those of perceptual networks (*p*<0.01, Bonferroni-corrected) (Fig. 4A). 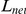, 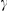, 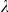 and 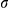 are also significantly different between groups (p<0.01, Bonferroni-corrected), the values of higher cognitive being larger than those of perceptual networks (Fig. 4B–E). Furthermore, we calculated network properties for RSNs with equalized node number (Fig. S3 and Table S1). Each normalized RSN exhibited small-world topology, confirming all results obtained from RSNs without the node-normalization procedure.

**Table 1:** Summary of network measures for each RSN.

RSN	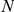	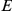 (mean±std)	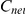 (mean±std)	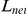 (mean±std)	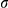 (mean±std)	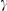 (mean±std)	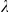 (mean±std)
CEN	2842	1.38e+6±0.35e+6	0.70±0.05	1.74±0.12	1.31±0.25★	1.37±0.28★	1.05±0.02⋆
DAN	3003	1.47e+6±0.36e+6	0.68±0.04	1.75±0.13	1.50±0.31★	1.58±0.37★	1.04±0.03⋆
DMN	7534	8.17e+6±2.07e+6	0.66±0.05	1.80±0.10	1.35±0.25★	1.42±0.28★	1.05±0.02⋆
SMN	1653	0.70e+6±0.20e+6	0.78±0.08	1.51±0.16	1.20±0.22★	1.21±0.23★	1.01±0.01⋆
AN	1980	1.01e+6±0.27e+6	0.76±0.07	1.50±0.15	1.18±0.19★	1.19±0.20★	1.01±0.01⋆
VN	1819	1.05e+6±0.24e+6	0.83±0.06	1.38±0.15	1.13±0.14★	1.14±0.15★	1.01±0.01⋆

N: number of voxels (nodes) in each RSN; E: number of edges in each RSN.

★: Significantly larger than 1 (one sample t-test, p<0.01, Bonferroni-corrected).

⋆: No significant difference compared to 1 (one sample t-test).

## Discussion

We investigated the network properties of six well-documented RSNs by combining ICA and graph theoretical analysis. Our results showed that each RSN had robust small-world properties, as evidenced by 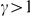 and 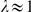 (Fig. 1 and Table 1). Both power in low-frequency band (between 0.01 and 0.08 Hz) and small-world index of the higher cognitive networks (CEN, DAN and DMN) were significantly greater than those of perceptual networks (AN, SMN and VN) (*p*<0.01, Bonferroni-corrected). For the first time, these findings provide quantitative evidence for the dichotomy between higher cognitive and perceptual networks [31], [36].

To define large-scale intrinsic brain networks we used ICA, a data-driven analysis. This approach is particularly valuable for the investigation of brain networks modulated by task performance [26], but also at rest [19], [23], [25]. The so-called resting state networks (RSNs) [21], [22] are supposed to reflect intrinsic energy demands and synchronizations of neuronal populations within a set of neuroanatomically and functionally organized brain regions [21], [55]. In this study, we focused on 3 perceptual (AN, SMN and VN) and 3 higher cognitive (CEN, DAN and DMN) networks to investigate similarities and differences in their network properties. The RSNs obtained from our data were consistent with previous resting-state fMRI studies [19], [22], [23], [25], [34].

In the power spectrum analysis, the higher cognitive networks exhibited significantly greater low-frequency power than the perceptual networks. This finding confirms the suggested dissociation between elementary level and higher level networks [31], [32], [37]. Furthermore, those findings support the concept that perceptual networks and higher cognitive networks are involved at different levels of functional processing [34], [36], with a different allocation of brain energy between the two groups. Not surprisingly, the DMN exhibited the largest low-frequency power among the others [18], [56], [57], [58] It is also possible that the DMN integrates information from other RSNs [34], which would also support the current findings.

All six RSNs were characterized by small-world topology [4], confirming and expanding findings from previous studies on the whole human brain network. The two key features of small-world topology, i.e. high clustering and short paths, reflect global and local parallel information processing [3]. Recently, small-world characteristics have been found in large-scale structural [59], [60] and functional brain networks [3], [61], and at a wide range of spatial and temporal scales. However, these studies mainly focused on whole-brain networks [5], [8], [10], [62], [63]. Exploring the topological attributes of specific RSNs may shed light on the architecture of the human brain. Small-world topology might reflect a principle of optimal architecture in the human brain [64]. Our data suggests that information is efficiently transferred not only in the whole brain network, but also within specific sub-networks, such as the RSNs.

We clearly found a difference in small-world characteristics between higher cognitive and perceptual networks, confirming our study hypothesis. Higher level cognitive networks, such as the DMN, DAN and CEN, are thought to occupy a different hierarchy in brain structure compared to perceptual networks [65]. In synaptic hierarchy, the lower synaptic levels mainly participate in encoding information from visual, auditory and somato-motor cortex, while the higher synaptic levels relate to cognitive processing, such as attention, emotion, working memory and mental imagery [31]. In addition, previous works have revealed that higher cognitive networks mature through ontogeny, while sensory networks were well-developed in early childhood [32], [66], [67].

Previous studies suggested competition between RSNs, as for example between the task-negative network (i.e. DMN) and task-positive networks [46]. To better understand the dissociation found between higher cognitive and perceptual networks, we examined the presence of competition by correlation analysis (see Text S2). Our data showed anti-correlations between these two network groups (Fig. S4A). By further exploring the correlations among RSNs, we also found selective differences, and the presence of competition within groups. For instance, the DMN was negatively correlated with the CEN, DAN and the perceptual networks (Fig. S4B), as already suggested by previous studies [46], [68], [69]. Taken together, the correlation results further demonstrate the fractionation between perceptual and higher cognitive networks, and suggest the presence of competition processes between the two network groups. Further work will be necessary to elucidate mechanisms of competition between RSNs, and how they relate to topological properties.

The main limitation of the present study is related to the spatial resolution and signal-to-noise ratio of the functional images, which may have an effect on the network topology measures. In this regard, we focus on two methodological considerations in particular. First, we collected the fMRI data at about 4-mm resolution, and resliced them to 3-mm cubic voxels after non-linear spatial transformation to a template space (MNI space). For subjects' with brain larger than the standard template, the normalization procedure deforms the original sampling grid at 4 mm to an inhomogeneous grid with resolution locally below 4 mm. We therefore resampled our data at 3 mm isotropic to preserve the spatial specificity of our data, as suggested previously [70], [71]. Importantly, the resampling at a resolution lower than the native one does not induce a loss of information, in compliance with the Nyquist–Shannon sampling theorem. Second, we applied spatial smoothing before network construction, to reduce noise in the functional images. It has been demonstrated that, for anatomically-defined areas, spatial smoothing may introduce (local) artificial correlations between ROIs [10], [11]. In this study, however, we calculated network properties at a voxel level, using the spatial extent of ICA-based RSNs to define functional ROIs. So far, no study has examined whether smoothing could introduce artificial connectivity at the network level [72]. For this reason, we examined the network topological properties in RSNs calculated without the smoothing step. Importantly, we found results very similar to those with smoothing (Fig. S5). We concluded that, in the present study, spatial smoothing had only minimal influence on RSN network construction.

In summary, we robustly found small-world properties in RSNs. For the first time, we showed quantitative evidence for the topological fractionation between perceptual and higher cognitive networks. This suggests that RSNs may occupy different hierarchical levels within the intrinsic functional architecture of the human brain. Our approach to investigate topological properties in RSNs may be extended to clinical research, especially to diseases that show selective abnormal connectivity in specific brain networks.

## Supporting Information

Text S1 **Reliability test.**
(DOC)

Text S2 **Correlation matrix between perceptual and higher cognitive networks.**
(DOC)

Figure S1 **Fractional amplitude of low-frequency fluctuation (fALFF) for each RSN.** Fractional ALFF values were defined as the ratio of total power within the low-frequency range (0.01–0.08 Hz) to that of the entire detectable frequency range. fALFF can provide specific measure of low-frequency spontaneous fluctuations in the BOLD signal. The vertical coordinates indicate the value of fALFF for each RSN. Error bars correspond to SD. We found that higher cognitive networks (CEN, DAN and DMN) exhibited higher fALFF values than perceptual networks (SMN, AN and VN) (two-way ANOVA, p = 0.0153).
(TIF)

Figure S2
**Mean path length and clustering coefficient for each RSN with equalized node number.** (A) Mean shortest path length, 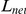, and (B) clustering coefficient, 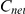, for each RSN as a function of correlation threshold 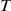 (0.125–0.55). Error bars correspond to SEM.(TIF)

Figure S3
**Statistical analysis of network properties for higher cognitive and perceptual networks after the node-normalization procedure.** The group of higher cognitive networks includes DMN, DAN and CEN; the group of perceptual networks includes SMN, VN and AN. Binary graphs were calculated using the correlation threshold 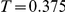. (A) Mean clustering coefficient, 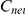; (B) shortest path length, 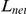; (C) small-world index, 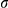; (D) normalized clustering coefficient, 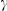; (E) normalized shortest path length, 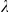. Error bars correspond to SD. Asterisks indicate statistically significant differences between the two network groups (two-way ANOVA, p<0.01, Bonferroni-corrected).(TIF)

Figure S4 **Correlation matrix of RSN time-courses.** The mean correlation matrix obtained by averaging a set of correlation matrices across subjects between the two network groups (higher cognitive and perceptual networks, respectively) (A), and among the six RSNs (B). Higher cognitive networks were significantly anti-correlated to perceptual networks. In line with previous findings, DMN (task-negative) was negatively correlated to other cognitive (task-positive) networks (i.e. CEN and DAN) and perceptual networks (SMN, AN and VN).
(TIF)

Figure S5
**Statistical analysis of network properties for higher cognitive and perceptual networks, defined without using spatial smoothing.** The group of higher cognitive networks includes DMN, DAN and CEN; the group of perceptual networks includes SMN, VN and AN. Binary graphs were calculated using the correlation threshold 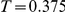. (A) Mean clustering coefficient, 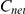; (B) shortest path length, 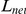; (C) small-world index, 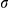; (D) normalized clustering coefficient, 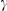; (E) normalized shortest path length, 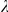. Each RSN had robust small-world properties, as evidenced by 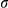 and 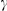 significantly larger than 1 (C and D), and 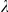 not different from 1 (*p*<0.01, Bonferroni-corrected) (E) for each RSN. Error bars correspond to SD. Asterisks indicate statistically significant differences between the two network groups (two-way ANOVA, p<0.01, Bonferroni-corrected).(TIF)

Table S1 **Summary of network measures for each RSN after the node-normalization procedure.**
(DOC)
