# Supplementary material for: Topological Fractionation of Resting-State Networks
Source: PLoS One. 2011 Oct 19;6(10):e26596. doi: 10.1371/journal.pone.0026596 (PMC3197522; doi:10.1371/journal.pone.0026596)
Supplement: Text S1 — Reliability test. (DOC) [file pone.0026596.s001.doc]

**Text-S1**

***Reliability test***

In the graph theoretical analysis, the presence of RSNs with different number of nodes may in principle result in the detection of different network topological properties. To eliminate this potential confound, we normalized the number of nodes across RSNs via a down-sampling procedure. First, we selected the RSN with the minimum number of nodes among RSNs (i.e. SMN), namely reference RSN. Next, we randomly selected nodes in a given RSN by sampling without replacing, for a number of nodes equal to that in the reference RSN. We applied this procedure to the remaining five RSNs. In this way, each RSN was considered with identical node number. For each RSN, network topological properties were re-analyzed and compared with the properties inferred without node-number normalization.
