# Supplementary material for: Topological Fractionation of Resting-State Networks
Source: PLoS One. 2011 Oct 19;6(10):e26596. doi: 10.1371/journal.pone.0026596 (PMC3197522; doi:10.1371/journal.pone.0026596)
Supplement: Text S2 — Correlation matrix between perceptual and higher cognitive networks. (DOC) [file pone.0026596.s002.doc]

**Text-S2**

***Correlation matrix between perceptual and higher cognitive networks***

Given the observed dichotomy between topological properties of perceptual (SMN, AN, and VN) and higher cognitive networks (DMN, CEN, and DAN), we examined the correlations between their time-courses. First, we created two masks to select voxels belonging to the two network groups, respectively. Next, we extracted two representative group time-courses by averaging across voxels in the masks. Finally, we calculated the Pearson correlation coefficient between the two time-courses. To further explore the correlations between the six RSNs, we also extracted RSN time-courses by averaging across voxels belonging each RSN separately, and we calculated a cross-correlation matrix (dimension $6\times6$).
