# Supplementary figures and images for: Topological Fractionation of Resting-State Networks
Source: PLoS One. 2011 Oct 19;6(10):e26596. doi: 10.1371/journal.pone.0026596 (PMC3197522; doi:10.1371/journal.pone.0026596)

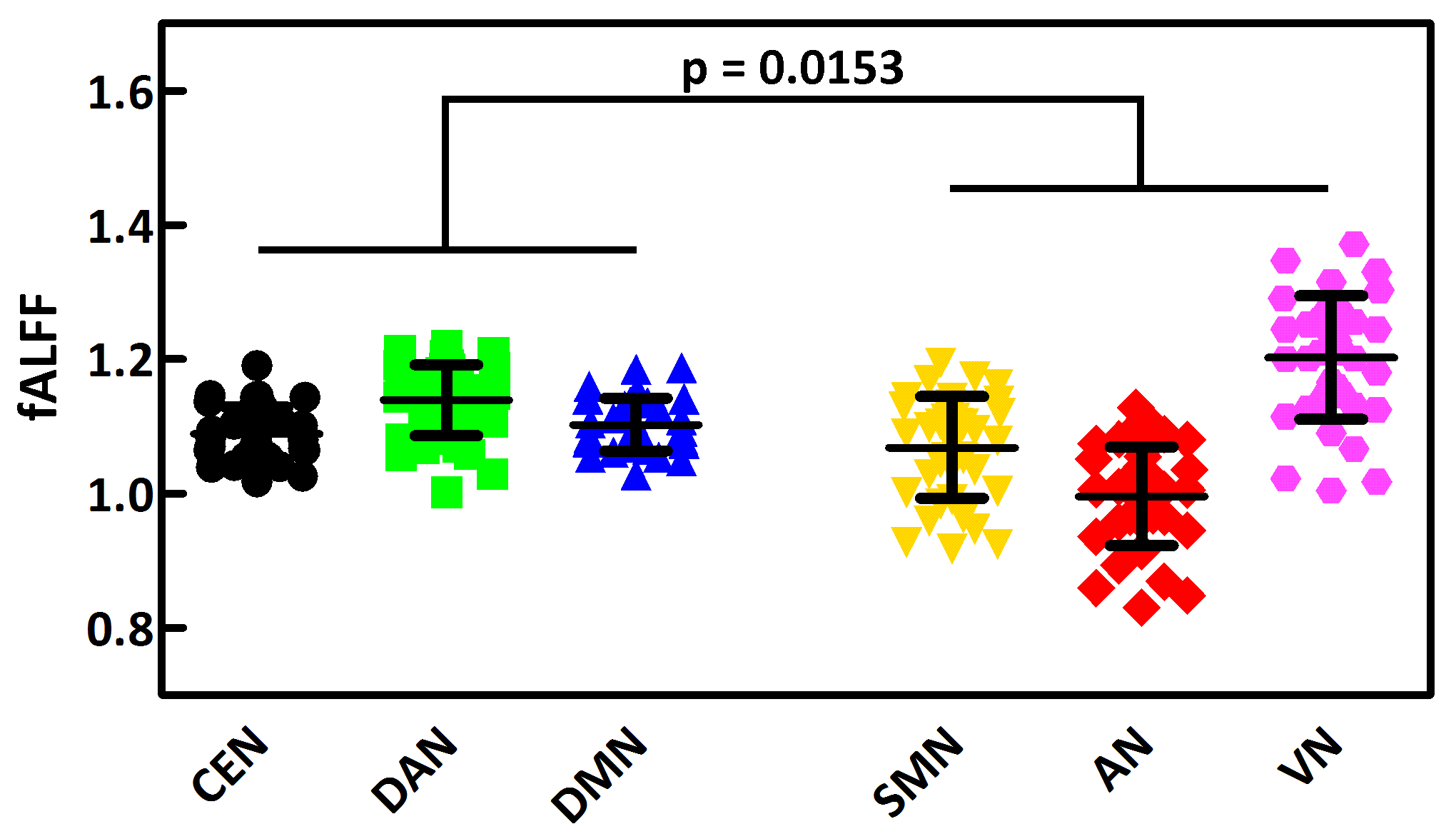

Supplement: Figure S1 — Fractional amplitude of low-frequency fluctuation (fALFF) for each RSN. Fractional ALFF values were defined as the ratio of total power within the low-frequency range (0.01–0.08 Hz) to that of the entire detectable frequency range. fALFF can provide specific measure of low-frequency spontaneous fluctuations in the BOLD signal. The vertical coordinates indicate the value of fALFF for each RSN. Error bars correspond to SD. We found that higher cognitive networks (CEN, DAN and DMN) exhibited higher fALFF values than perceptual networks (SMN, AN and VN) (two-way ANOVA, p = 0.0153). (TIF) [file pone.0026596.s003.tif]

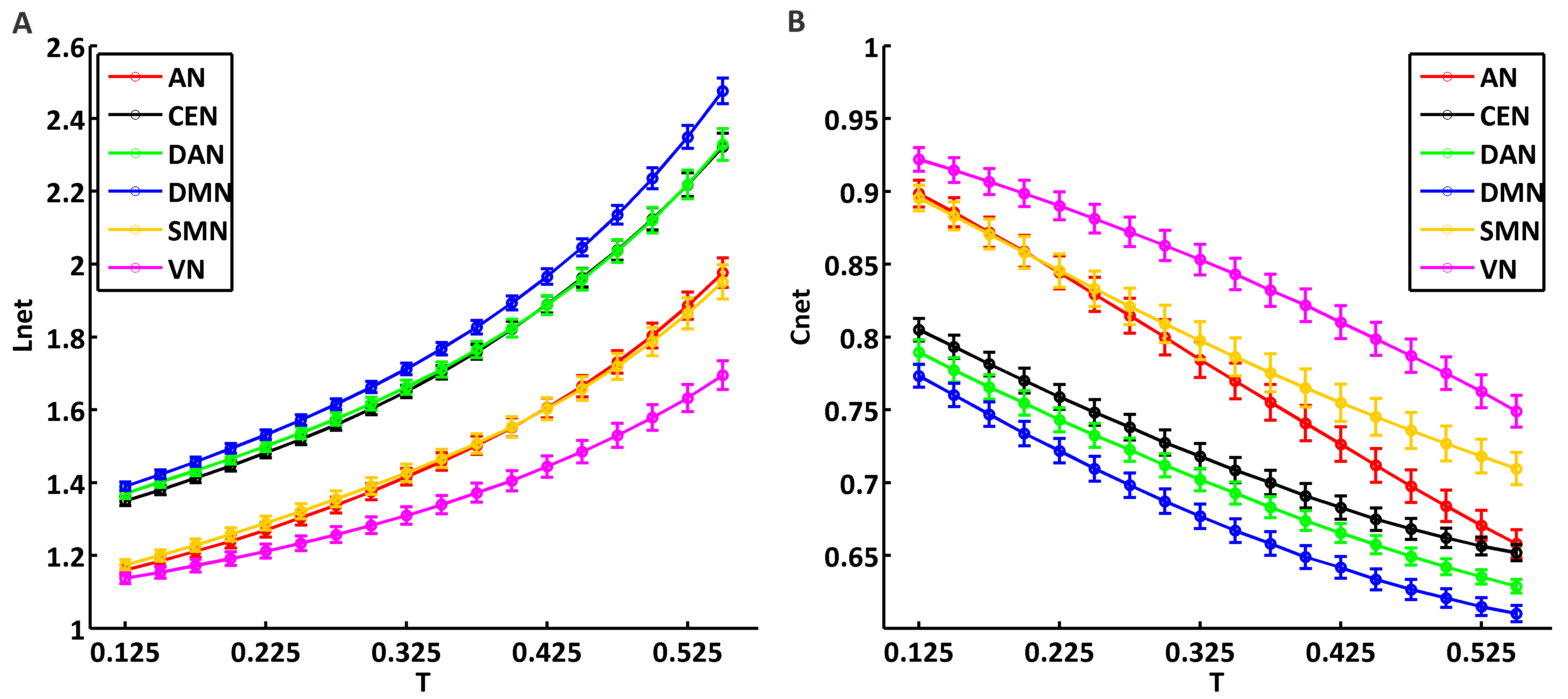

Supplement: Figure S2 — Mean path length and clustering coefficient for each RSN with equalized node number. (A) Mean shortest path length, , and (B) clustering coefficient, , for each RSN as a function of correlation threshold (0.125–0.55). Error bars correspond to SEM. (TIF) [file pone.0026596.s004.tif]

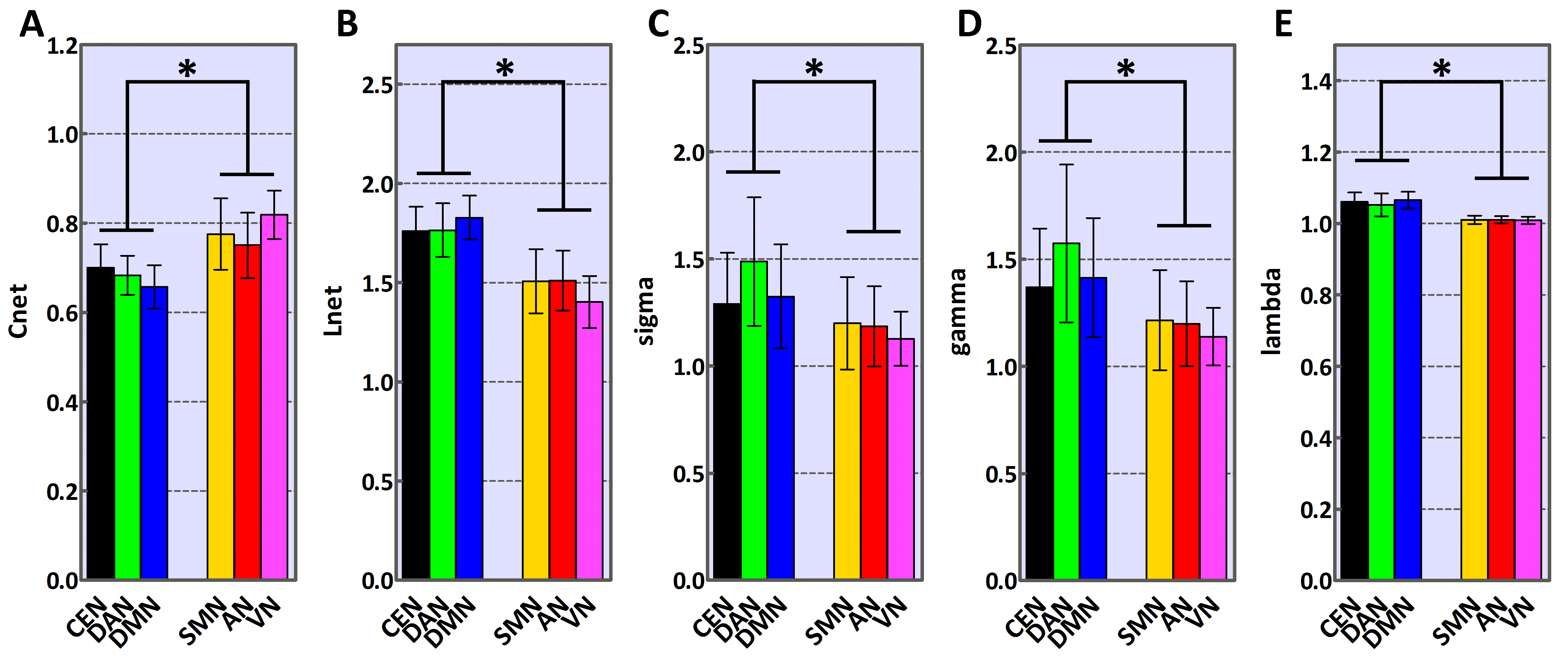

Supplement: Figure S3 — Statistical analysis of network properties for higher cognitive and perceptual networks after the node-normalization procedure. The group of higher cognitive networks includes DMN, DAN and CEN; the group of perceptual networks includes SMN, VN and AN. Binary graphs were calculated using the correlation threshold . (A) Mean clustering coefficient, ; (B) shortest path length, ; (C) small-world index, ; (D) normalized clustering coefficient, ; (E) normalized shortest path length, . Error bars correspond to SD. Asterisks indicate statistically significant differences between the two network groups (two-way ANOVA, p<0.01, Bonferroni-corrected). (TIF) [file pone.0026596.s005.tif]

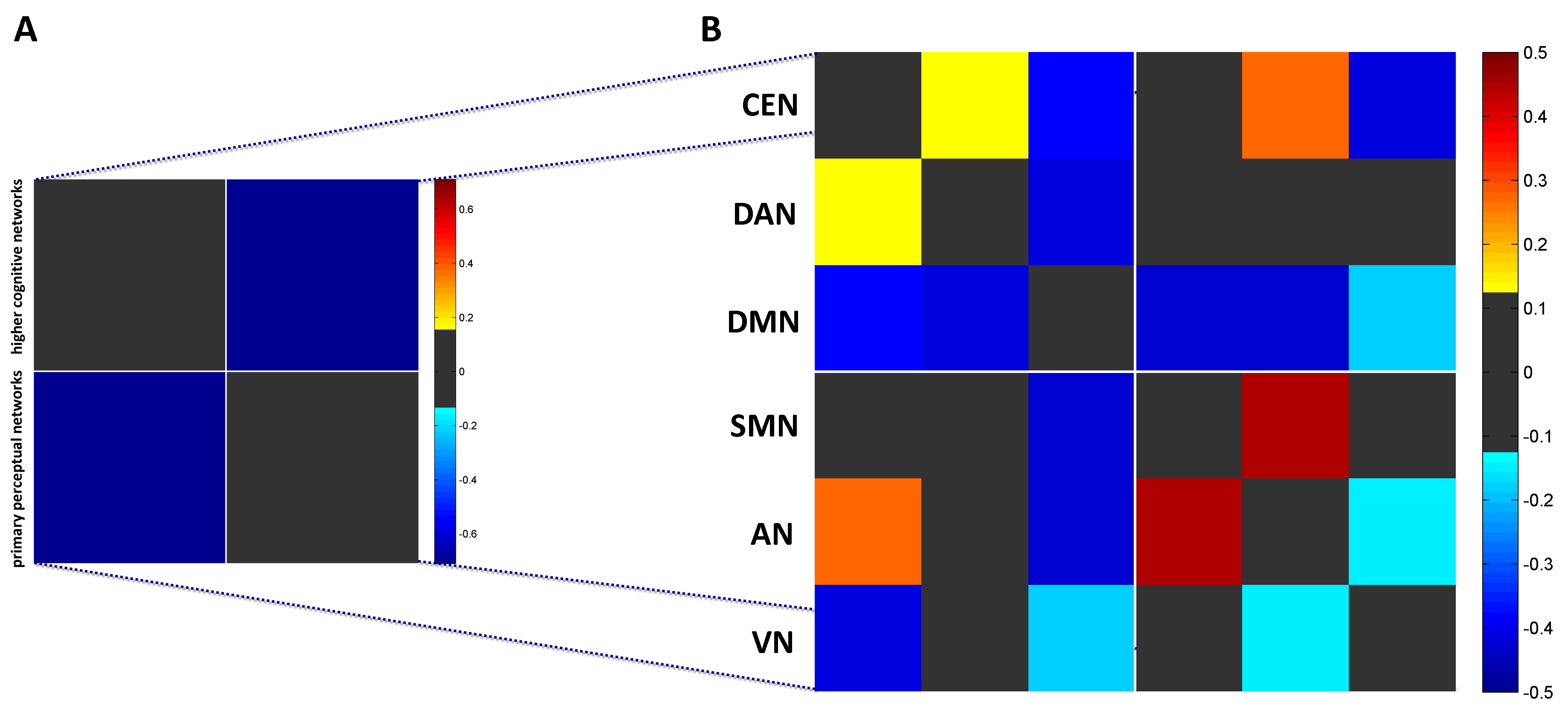

Supplement: Figure S4 — Correlation matrix of RSN time-courses. The mean correlation matrix obtained by averaging a set of correlation matrices across subjects between the two network groups (higher cognitive and perceptual networks, respectively) (A), and among the six RSNs (B). Higher cognitive networks were significantly anti-correlated to perceptual networks. In line with previous findings, DMN (task-negative) was negatively correlated to other cognitive (task-positive) networks (i.e. CEN and DAN) and perceptual networks (SMN, AN and VN). (TIF) [file pone.0026596.s006.tif]

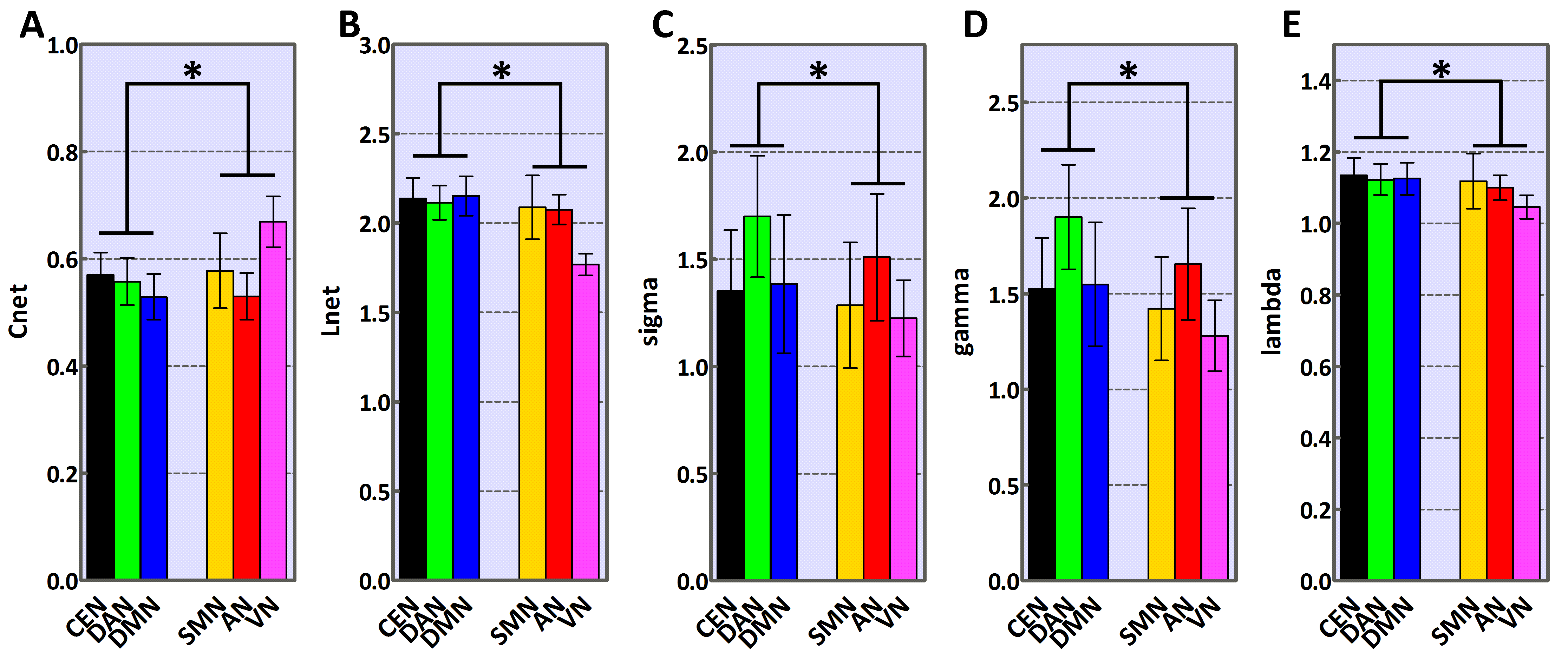

Supplement: Figure S5 — Statistical analysis of network properties for higher cognitive and perceptual networks, defined without using spatial smoothing. The group of higher cognitive networks includes DMN, DAN and CEN; the group of perceptual networks includes SMN, VN and AN. Binary graphs were calculated using the correlation threshold . (A) Mean clustering coefficient, ; (B) shortest path length, ; (C) small-world index, ; (D) normalized clustering coefficient, ; (E) normalized shortest path length, . Each RSN had robust small-world properties, as evidenced by and significantly larger than 1 (C and D), and not different from 1 (p<0.01, Bonferroni-corrected) (E) for each RSN. Error bars correspond to SD. Asterisks indicate statistically significant differences between the two network groups (two-way ANOVA, p<0.01, Bonferroni-corrected). (TIF) [file pone.0026596.s007.tif]
