# Supplementary material for: Topological Fractionation of Resting-State Networks
Source: PLoS One. 2011 Oct 19;6(10):e26596. doi: 10.1371/journal.pone.0026596 (PMC3197522; doi:10.1371/journal.pone.0026596)
Supplement: Table S1 — Summary of network measures for each RSN after the node-normalization procedure. (DOC) [file pone.0026596.s008.doc]

**Table S1. Summary of network measures for each RSN** after the node-normalization procedure

| RSN | $N$ | $E$ (mean$\pm$std) | $C_{net}$ (mean$\pm$std) | $L_{net}$ (mean$\pm$std) | $\sigma$ (mean$\pm$std) | $\gamma$ (mean$\pm$std) | $\lambda$ (mean$\pm$std) |
| --- | --- | --- | --- | --- | --- | --- | --- |
| CEN | 1653 | 4.67e+5$\pm$1.18e+5 | 0.70±0.05 | 1.76±0.12 | 1.29±0.24★ | 1.37±0.27★ | 1.06±0.03☆ |
| DAN | 1653 | 4.43e+5$\pm$1.10e+5 | 0.68±0.04 | 1.77±0.14 | 1.49±0.30★ | 1.57±0.37★ | 1.05±0.03☆ |
| DMN | 1653 | 3.93e+5$\pm$0.99e+5 | 0.66±0.05 | 1.83±0.11 | 1.32±0.24★ | 1.41±0.28★ | 1.07±0.02☆ |
| SMN | 1653 | 6.95e+5$\pm$2.00e+5 | 0.78±0.08 | 1.51±0.16 | 1.20±0.22★ | 1.21±0.23★ | 1.01±0.01☆ |
| AN | 1653 | 6.91e+5$\pm$1.88e+5 | 0.75±0.07 | 1.51±0.15 | 1.19±0.19★ | 1.20±0.20★ | 1.01±0.01☆ |
| VN | 1653 | 8.33e+5$\pm$1.65e+5 | 0.82±0.05 | 1.40±0.13 | 1.13±0.13★ | 1.14±0.13★ | 1.01±0.01☆ |

N: number of voxels (nodes) in each RSN; E: number of edges in each RSN;

★: Significantly larger than 1 (one sample t-test, p < 0.01, Bonferroni-corrected);

☆: No significant difference compared to 1 (one sample t-test).
